# Isolation and characterization of a novel plasma membrane protein, osteoblast induction factor (obif), associated with osteoblast differentiation

**DOI:** 10.1186/1471-213X-9-70

**Published:** 2009-12-21

**Authors:** Takashi Kanamoto, Koji Mizuhashi, Koji Terada, Takashi Minami, Hideki Yoshikawa, Takahisa Furukawa

**Affiliations:** 1 Department of Developmental Biology, Osaka Bioscience Institute, 6-2-4 Furuedai, Suita, Osaka 565-0874, Japan; 2 Department of Orthopedic Surgery, Graduate School of Medicine, Osaka University, Osaka 565-0871, Japan; 3 Department of Molecular and Vascular Medicine, The Research Center for Advanced Science and Technology, University of Tokyo, Tokyo 153-8904, Japan

## Abstract

**Background:**

While several cell types are known to contribute to bone formation, the major player is a common bone matrix-secreting cell type, the osteoblast. Chondrocytes, which plays critical roles at several stages of endochondral ossification, and osteoblasts are derived from common precursors, and both intrinsic cues and signals from extrinsic cues play critical roles in the lineage decision of these cell types. Several studies have shown that cell fate commitment within the osteoblast lineage requires sequential, stage-specific signaling to promote osteoblastic differentiation programs. In osteoblastic differentiation, the functional mechanisms of transcriptional regulators have been well elucidated, however the exact roles of extrinsic molecules in osteoblastic differentiation are less clear.

**Results:**

We identify a novel gene, *obif *(*osteoblast induction factor*), encoding a transmembrane protein that is predominantly expressed in osteoblasts. During mouse development, *obif *is initially observed in the limb bud in a complementary pattern to *Sox9 *expression. Later in development, *obif *is highly expressed in osteoblasts at the stage of endochondral ossification. In cell line models, *obif *is up-regulated during osteoblastic differentiation. Exogenous *obif *expression stimulates osteoblastic differentiation and *obif *knockdown inhibits osteoblastic differentiation in preosteblastic MC3T3-E1 cells. In addition, the extracellular domain of obif protein exhibits functions similar to the full-length obif protein in induction of MC3T3-E1 differentiation.

**Conclusions:**

Our results suggest that *obif *plays a role in osteoblastic differentiation by acting as a ligand.

## Background

The skeleton is a multifunctional system with physiological roles in providing a rigid framework and support, acting as the primary storage site for mineral salts, and functioning in hematopoiesis. While several cell types are known to contribute to bone formation, the major player is a common bone matrix-secreting cell type, the osteoblast.

Chondrocytes, which play critical roles at several stages of endochondral ossification, and osteoblasts are derived from common precursors, and both intrinsic cues and signals from extrinsic cues play critical roles in the lineage decision of these cell types [1-3]. The targeted mutation of *Runx2 *or *Osterix*, transcription regulators highly expressed in osteoblast progenitors, result in the lack of mature osteoblasts, demonstrating that these factors are essential for osteoblastogenesis [4-7]. Several studies have shown that cell fate commitment within the osteoblast lineage requires sequential, stage-specific, *Ihh *and canonical Wnt/β-catenin signaling to promote osteoblastic, and also to block chondrogenic, differentiation programs [8-11]. Another recent report shows that the inhibitory effect of *Sox9 *on osteoblastic and chondrocyte maturation via repression of *Runx2 *function is an essential mechanism for osteochondroprogenitor cell fate determination [12].

Most bones are formed by endochondral ossification in which mesenchymal condensations differentiate into chondrocytes, forming a cartilaginous template prefiguring the future skeletal elements. The perichondrium and perioseum are generated around the nascent cartilage, and cells surrounding the zone of hypertrophic chondrocytes begin to differentiate into osteoblasts. *Sox9 *is expressed in all chondroprogenitors and chondrocytes except hypertrophic chondrocytes, and is required for the sequential steps of chondrogenesis before and after mesenchymal condensations [13,14].

Thus, in osteoblastic differentiation, the functional mechanisms of transcriptional regulators have been well elucidated, however the exact roles of extrinsic molecules in osteoblastic differentiation are less clear. In our microarray screening aiming to identify novel molecular pathways in chondrocyte differentiation, we identified a novel gene encoding a transmembrane protein, which is predominantly expressed in osteoblasts during mouse development. We named this gene *obif *(*osteoblast induction factor*) and performed functional analyses using cell culture models.

We found that *obif *overexpression in the preosteoblastic MC3T3-E1 cells induces differentiation and maturation of osteoblasts. Knockdown of *obif *suppresses osteoblastic differentiation of MC3T3-E1 cells. We also present data demonstrating that the extracellular domains of both mouse and human obif proteins augment osteoblastic differentiation-induction activity, suggesting that obif acts in a ligand-like manner. Together, our results suggest that *obif *is involved in the differentiation of osteoblasts through intercellular interaction during development.

## Results

### Identification and intracellular localization of obif protein

**Figure 1:**
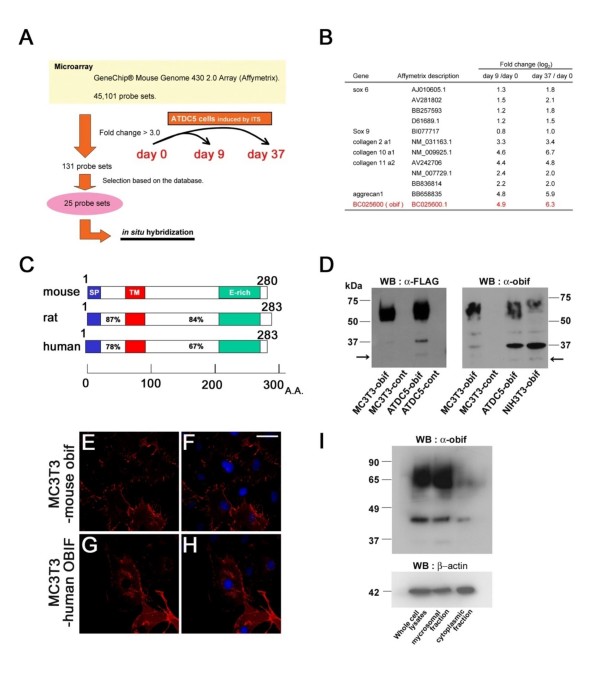
**Identification of a novel plasma membrane protein, obif, and its subcellular localization**. (A) The strategy of microarray-based screening to identify up-regulated genes in chondrocyte differentiations of ATDC5 cell line. (B) Fold changes of representative chondrocyte markers (black) and *obif *(red) transcripts which are up-regulated in the microarray analysis. (C) Schematic diagram of obif protein structure. The percent sequence identities of each region are shown: SP, signal peptide; TM, transmembrane domain; E-rich, glutamic acid-rich domain. Numbers represent amino acid residues. (D) Detection of FLAG-tagged obif protein. Whole cell lysates of MC3T3-obif, MC3T3-cont, ATDC5-obif, ATDC5-cont, and NIH3T3-obif were electrophoresed by SDS-PAGE. Immunoblots were probed with anti-FLAG M2 antibody or anti-obif antibody. Arrows represent the molecular size of obif protein predicted from its amino acid sequences (29.4 kDa). (E-H) Confocal analysis of overexpressed mouse and human obif in MC3T3-E1 cells. Cells expressing FLAG-tagged mouse obif were stained with anti-obif antibody (red) (E, F). Cells expressing FLAG-tagged human OBIF were stained with anti-FLAG M2 antibody (red) (G, H). Nuclei were stained with DAPI (blue). Scale bar = 50 μm. (I) Subcellular localization of obif protein. Subcellular fractions of ST2 cells expressing exogenous mouse obif were analyzed by Western blotting. Immunoblots were probed with an anti-obif antibody to localize obif (top) and with an antibody against β-actin, an abundant cytoplasmic protein (bottom).

With the aim of identifying novel molecules involved in chondrocyte differentiation, we performed a microarray screening using a mouse chondroprogenitor cell line, ATDC5 (Figure 1A). We extracted total RNA at day 0 (the confluence stage), day 9 (the cartilage nodule formation stage), and day 37 (the cellular hypertrophy and calcification stage). With the gene expression profile analysis, we identified 131 probe sets that showed more than a three-fold change in expression level at day 9 or day 37 relative to day 0. From among these genes, we selected 25 genes that have not as yet been known to be associated with chondrogenesis and osteoblastogenesis, and further investigated their expression patterns. We found one gene that shows an expression pattern predominantly in the skeletal systems of mouse embryos and up-regulation upon differentiation in ATDC5 cells. On the basis of our functional assay, we named this gene *obif *(*osteoblast induction factor*) (GenBank TPA #BC025600) (Figure 1B) which is the same gene with *transmembrane protein 119 *(*tmem119*).

The deduced amino acid sequence of mouse *obif *contains a signal sequence, a single transmembrane domain, and a glutamic acid-rich region. This gene is conserved among species from chicken to human (Figure 1C and Additional file 1). We also isolated a human *OBIF *cDNA from commercially available human fetal brain RNA by RT-PCR. The genomes of zebra fish and *Xenopus tropicalis *also contain sequences that are homologous to mouse *obif *(data not shown). We then examined the subcellular localization of obif. Using a retrovirus vector, we overexpressed a FLAG-tagged obif in various cell lines. To confirm the obif expression, we performed Western blot analysis using anti-FLAG M2 antibody and the antibody that we raised against obif (Figure 1D). With both of these antibodies, we detected a strong band of approximately 37 kD, a weak band of approximately 44 kD, and broad bands of 50 - 75 kD in all cell lines in which obif was overexpressed (Figure 1D). Using the on-line predictor method NetOGlyc 3.1 [15], we found several potential O-glycosylation sites between the signal sequence and transmembrane domain of obif (Additional file 1). This observation strongly suggests that obif is a type Ia transmembrane protein in which the N-terminal region is frequently modified post-transcriptionally. Next, we examined the intracellular localization of obif using MC3T3-E1 cells stably overexpressing mouse obif (MC3T3-obif) or human OBIF (MC3T3-OBIF) by immunostaining (Figures 1E-H). Obif/OBIF are mainly localized to the plasma membrane. This observation is coincident with the result of Western blot analysis on the subcellular fractionated samples (Figure 1I), in which the microsomal fraction contained an abundant amount of obif compared with the cytoplasmic fraction.

### Obif transcript is expressed in osteoblast-lineage cells

**Figure 2:**
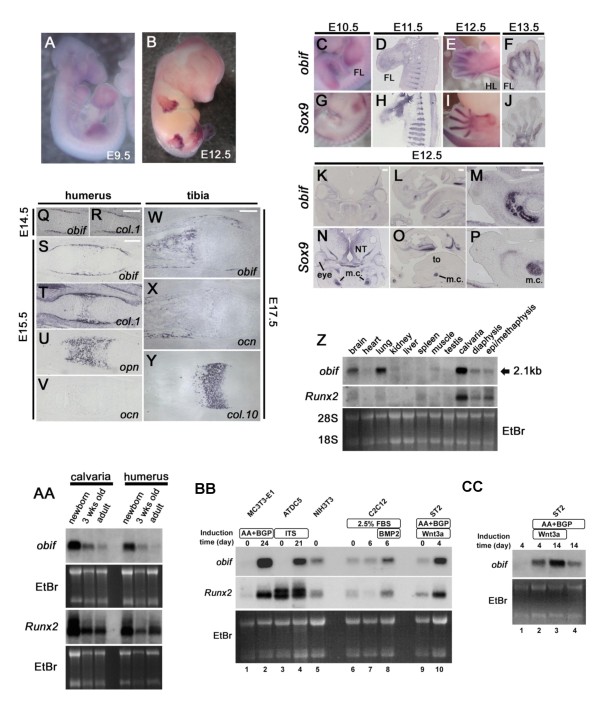
**Predominant expression of *obif *transcript in osteoblast-lineage cells**. (A, B) Expressions of *obif *in mouse embryos examined by whole mount *in situ *hybridization. (C-P) *In situ *hybridization analysis of mouse *obif *and *Sox9 *in the developing mouse limb, rib, and mandible. *Obif *expression was complementary to that of *Sox9 *in the developing limb bud (C-J), rib (D, H) and mandible (K-P). FL, forelimb; HL, hindlimb; NT, neural tube; to, tongue; m.c., Meckel's cartilage. Scale bars = 200 μm. (Q-Y) Section *in situ *hybridization of mouse *obif *in endochondral ossification. At E14.5 *obif *transcripts were detected in the perichondrium but were absent from more centrally located chondrocytic cells (Q, R). In E15.5 humerus and E17.5 tibia, *obif *expression was found in cells associated with bone trabeculae and in cells associated with the formation of bone collars (S-Y). Scale bars = 200 μm. (Z) Northern blot analysis of *obif *and *Runx2 *expression in 4 week-old mouse tissues. The arrow corresponds to a 2.1 kb *obif *transcript. (AA) Northern blot analysis of *obif *and *Runx2 *expression in the calvaria and humeri harvested from newborn, 3 week-old, and adult mice. (BB, CC) Northern blot analysis of *obif *and *Runx2 *transcripts in cell differentiation models of osteoblasts and chondrocytes. Total RNAs, extracted from MC3T3-E1 cells (BB: lane 1, undifferentiated cells; lane 2, cells cultured in differentiation medium for 24 days), ATDC5 cells (BB: lane 3, undifferentiated cells; lane 4, cells cultured in differentiation medium for 21 days), NIH3T3 cells (BB, lane 5), C2C12 cells (BB: lane 6, undifferentiated cells; lane 7, cells cultured in myogenic differentiation medium for 6 days; lane 8, cells cultured in osteoblastic differentiation medium for 6 days), and ST2 cells (BB: lane 9, cells cultured in growth medium; lane 10, cells cultured in medium supplemented with Wnt3a and ascorbic acid for 4 days; and CC: lane 1, cells cultured in growth medium for 4 days; lane 2, cells cultured in medium with Wnt3a and ascorbic acid for 4 days; lane 3, cells cultured in medium with Wnt3a and ascorbic acid for 14 days; lane 4, cells cultured in medium with ascorbic acid for 14 days) were used for Northern blot analysis.

Early mouse embryos were examined for *obif *transcript expression by whole mount *in situ *hybridization (Figures 2A and 2B). No clear hybridization signal was detected at E8.5 (data not shown). At E9.5, the signal was seen in the whole limb buds (Figure 2A). At E12.5, the stage the cartilage primordiums were formed in the limb buds, *obif *transcripts were observed in the areas between these primordiums (Figure 2B).

We then compared the expression of *obif *with that of the chondrocytic marker *Sox9 *(Figures 2C-P). In the forelimb bud, *obif *is expressed in a broad region at E9.5 (Figure 2A). At E10.5 and E11.5, the signal is down-regulated in the proximal region where mesenchymal cells condense and differentiate into chondrocytes, as evidenced by the onset of *Sox9 *expression (Figures 2C, D, G, and 2H). At E12.5 and E13.5, while *Sox9 *shows a prominent signal in future digit regions, the expression of *obif *is most abundant in the interdigital mesenchyme (Figures 2E, F, I, and 2J). As in developing limb buds, *obif *expression is complementary to that of *Sox9 *in other tissues (Figures 2D, H, K-P). For instance, *obif *is expressed in cells between the condensations that form the future ribs at E11.5 (Figure 2D) and in the osteogenic mesenchyme surrounding Meckel's cartilage at E12.5 (Figures 2K-M). However, we could not detect *obif *transcripts in the chondrocyte-lineage cells such as the cartilage condensation and Meckel's cartilage where *Sox9 *is expressed (Figures 2C-P).

To investigate the *obif *transcript expression at later embryonic stages, we performed section *in situ *hybridization with several markers pertaining to endochondral ossification (Figures 2Q-Y). *Collagen1*(*Col.1*) and *osteocalcin (ocn) *are markers for mature osteoblasts. *Osteopontin *(*opn*) is a marker for both terminally differentiated chondrocytes and osteoblasts, and *collagen 10 *(*col.10*) is a marker for hypertrophic chondrocytes. Interestingly, the expression of *obif *was observed in the osteoblasts. In E14.5 humerus, *obif *transcripts were detected in the perichondrium of the hypertrophic chondrocyte region where *collagen1 *is expressed, but were absent from more centrally located chondrocytic cells (Figures 2Q and 2R). At E15.5, when *ocn *is not yet significantly expressed, *obif *is detected both in the prospective bone collar region marked with *collagen1 *and in the developing bone trabeculae marked with *opn *(Figures 2S-V). At E17.5, *obif *expression overlaps with *ocn *but not with *collagen10 *(Figures 2W-Y).

Next, we examined the expression of *obif *and *Runx2 *in various tissues of 4 week-old mice by Northern blot analysis (Figure 2Z). A 2.1 kb band was detected in the tissues containing osteoblasts (calvaria, long bones) and other tissues such as the brain and lung. The calvaria band was significantly stronger as compared with that of the brain or lung. High levels of *obif *expression at embryonic stages followed by decreased expression of *obif *at early postnatal stages suggest that *obif *works in both embryonic skeletal development and postnatal differentiation. During postnatal development, expression in calvaria and long bones decreases whereas *Runx2 *expression is comparably maintained (Figure 2AA), suggesting that *obif *does not have an important role in mature adult tissues.

### Obif expression is up-regulated during osteoblastic differentiation in cell line models

Several cell lines derived from mesenchymal tissues have been widely used to recapitulate *in vivo *cellular differentiation [16-20]. We used preosteoblastic MC3T3-E1 cells, myogenic C2C12 cells, and stromal ST2 cells as osteoblast differentiation models, and chondrogenic ATDC5 cells as a chondrocyte differentiation model. Initially, the endogenous *obif *in these cells was examined in comparison with *Runx2 *by Northern blot analysis (Figure 2BB). In undifferentiated MC3T3-E1 cells only a faint signal was detected, however, upon induction by ascorbic acid (AA) and β-glycerophosphate (BGP), *obif *transcripts dramatically increased (Figure 2BB: lanes 1 and 2). Similarly, although ATDC5 cells express almost no *obif *transcript in an undifferentiated state, *obif *expression is markedly up-regulated during differentiation induced by ITS (insulin/transferrin/sodium selenite) (Figure 2BB: lanes 3 and 4). NIH3T3 cells express a low amount of *obif *and *Runx2 *transcripts (Figure 2BB: lane 5). In C2C12 cells, *obif *expression increased during osteoblastic differentiation induced by BMP2 (Figure 2BB: lanes 6-8). In ST2 cells, *obif *expression increased during differentiation induced by ascorbic acid or Wnt 3a (Figure 2BB: lanes 9 and 10, and Figure 2CC: lanes 1-4).

### Endogenous obif protein is expressed in osteoblast-lineage cells

**Figure 3:**
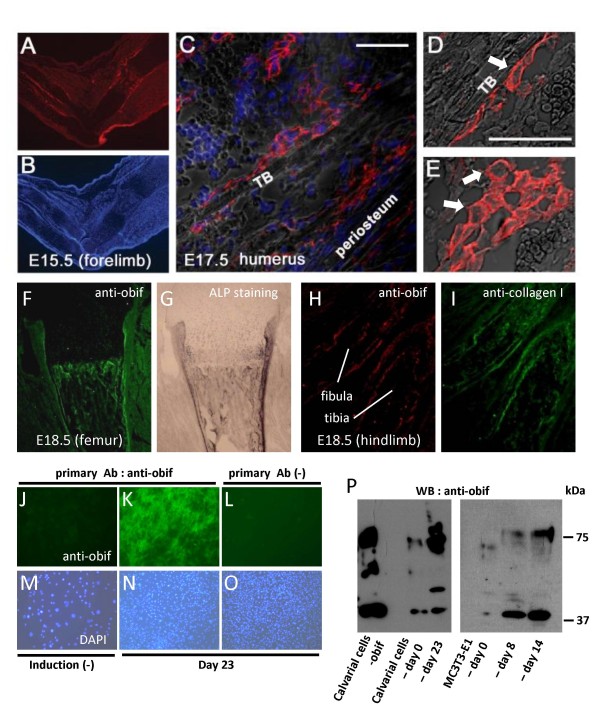
**Predominant expression of obif protein in osteoblast-lineage cells**. (A-E) Immunostaining of developing limbs using anti-obif antibody. Sections of limbs were stained with anti-obif antibody (red) and nuclei were stained with DAPI (blue). E15.5 forelimb sections (A, B). Scale bars = 200 μm. E17.5 humerus sections (C). *Obif *was expressed in spindle-shaped osteoblasts lining trabecular bone surfaces and in cuboidal-shaped osteoblasts. Scale bars = 50 μm. Higher magnification of E17.5 humerus sections shows that obif is localized to the plasma membrane (D, E). White arrows indicate typical cells that show the transmembrane pattern. (F, G) E 18.5 femur section was first stained with anti-obif antibody (green) and then stained for ALP. (H, I) E 18.5 hindlimb section was double-stained with anti-obif antibody (red) and anti-collagen I antibody (green). (J-O) Primary calvarial cells cultured in 12-well plates were immunostained with anti-obif antibody (J-L) and DAPI (M-O). Sparsely cultured cells (J, M). Densely cultured cells were incubated in presence of ascorbic acid for 23 days and used for analysis (K-O). L & O are negative controls. (P) Detection of endogenous obif protein. Whole cell lysates of calvarial cells-*obif *(stably infected), calvarial cells-day 0 (not stimulated), calvarial cells-day 23 (stimulated for 23 days), MC3T3-E1 cells-day 0 (not stimulated), MC3T3-E1 cells-day 8 (stimulated for 8 days), and MC3T3-E1 cells-day 14 (stimulated for 14 days) were electrophoresed by SDS-PAGE. Immunoblots were probed with the anti-obif antibody. Calvarial cells-*obif *is a positive control.

In order to investigate the localization of endogenous obif protein, we immunostained sections of embryonic forelimb and humerus using the anti-obif antibody (Figures 3A-E). Signals were observed in the perichondrium of the hypertrophic chondrocyte region, peripheral layers of the endochondral skeletal elements, and cells associated bone trabeculae including both the spindle-shaped osteoblasts and the cuboidal-shaped osteoblasts. In addition, we observed that *obif *appears to be expressed in the plasma membrane of osteoblasts (Figures 3C and 3E). To verify the expression of obif protein in osteoblast-lineage cells, we performed double staining of the developing limb using the obif antibody with other osteoblastic markers such as alkaline-phosphatase (ALP) and collagen 1 (Figures 3F-I). At late embryonic stages, co-localization of obif with ALP or with collagen type I was observed in the femur (Figures 3F and 3G) and hindlimb (Figures 3H and 3I).

Next, we examined obif protein expression in primary cultures of fetal mouse calvaria (Figures 3J-O). When calvarial cells were sparsely cultured, no obvious obif signals were observed (Figures 3J and 3M). On the other hand, when densely cultured cells were stimulated with osteoblastic differentiation medium, strong obif expression was detected (Figures 3K and 3N). By western blot analysis using primary culture cells and MC3T3-E1 cells, the elevation of the protein level was observed during calvarial cell maturation (Figure 3P).

### The role of obif in osteoblastogenesis

We then performed cell differentiation analysis using the retrovirus vector. For the overexpression study, cells were infected with retroviruses expressing both obif and GFP through IRES or expressing GFP only as a control. The infection efficiency was monitored by GFP expression (Additional file 2A, D, G, and 2J). Obif expression was confirmed by immunocytochemistry (Additional file 2C and 2I) and Western blot analysis using anti-obif antibody (Figure 1D). These results show that cultured cells can be infected efficiently (>95%) with retroviruses. These infected cells express GFP and/or obif protein even after extensive passaging. For gene knockdown, we made retroviruses expressing short hairpin RNAs (shRNAs) against *obif*. First, we designed three different siRNAs (siRNA-1, siRNA-2, and siRNA-3) and examined their knockdown effects (Additional file 3). MC3T3-obif cells and ATDC5-obif cells were transfected with siRNA-1, siRNA-2, siRNA-3, or control siRNA and analyzed by Western blotting. Non-transfected MC3T3-obif cells and ATDC5-obif cells were also electrophoresed. All of the siRNAs against *obif *showed strong knockdown effects (Additional file 3). Next, we made retroviral constructs overlapping the effective siRNA sequences, a control shRNA (sh-cont), sh292 which sequence overlaps siRNA-1 and siRNA-2 sequences, and sh301 which sequence overlaps siRNA-3 sequence. When infected, MC3T3-obif cells with sh292 retroviruses showed a strong knockdown effect and those with sh301 retroviruses showed a comparatively weak knockdown effect (Additional file 3). Using these retroviruses, we established cells stably expressing these shRNAs and performed knockdown studies.

**Figure 4:**
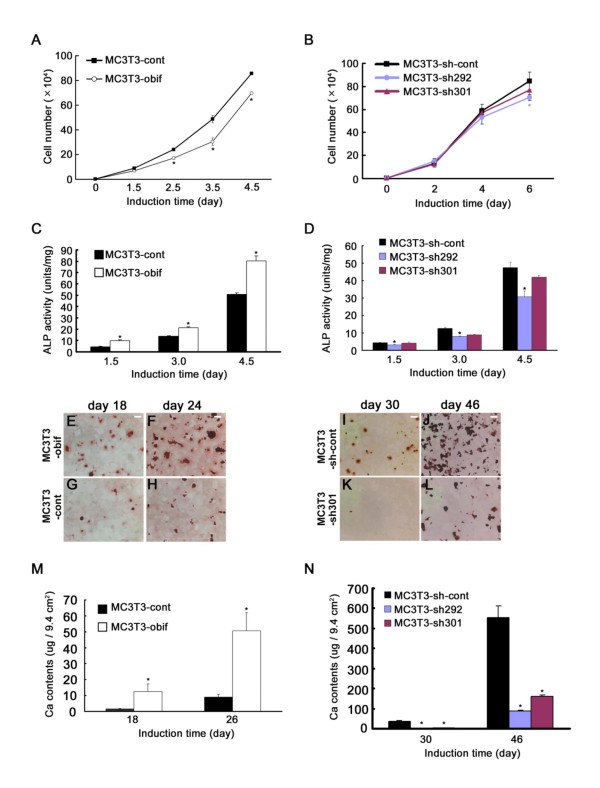
**The role of obif in the osteoblastic differentiation of MC3T3-E1 cells**. (A, B) Effect of *obif *expression on cell growth in the MC3T3-E1 cells. The proliferation rate decreased somewhat in MC3T3-obif compared with MC3T3-cont (A). Cells infected with retroviruses expressing shRNA against obif (MC3T3-sh292, MC3T3-sh301) showed almost the same level of cell growth with MC3T3-sh-cont (B). (C) Effect of obif expression on ALP activity in MC3T3-E1 cells cultured in the presence of ascorbic acid and β-glycerophosphate. (D) Effect of expression of shRNAs against obif in MC3T3-E1 cells. The reduction of *obif *expression caused a significant decrease of ALP activity at all time points. The decrease was proportionate to the strength of the shRNA's knockdown effects. (E-N) Mineral deposition visualized by Alizarin Red staining (E-L), and calcium contents measured by a colorimetric assay (M, N). The mineral deposition observed in MC3T3-obif cells and MC3T3-cont cells at days 18 and 24 (E-H). The mineral deposition observed in MC3T3-sh301 cells compared to MC3T3-sh-cont cells at days 30 and 46 (I-L). Scale bars = 2 mm. Calcium contents were significantly higher in MC3T3-obif cells than in MC3T3-cont cells both at day 18 and day 26 (M). Calcium deposition in MC3T3-sh292/301 and MC3T3-sh-cont cells at days 30 and 46 (N).

Initially, we examined the effect of *obif *overexpression on cell growth (Figures 4A and 4B). The proliferation rate decreased somewhat in MC3T3-obif cells compared with MC3T3-control (MC3T3-cont) cells (Figure 4A). The same effects of exogenous obif were observed in other cell lines, ATDC5, ST2, and C2C12 (data not shown). On the other hand, MC3T3-sh292 and -sh301 cells showed almost the same cell growth as cells infected with control retrovirus (MC3T3-sh-cont) (Figure 4B). This result indicates that effect of *obif *on cell differentiation described below is not mainly due to the effects on cell growth.

Next, we investigated the relationship between *obif *expression and osteoblast differentiation. We first examined an early stage osteoblastic marker, ALP activity. The retrovirally infected cells were cultured in osteoblast differentiation media and harvested at the indicated time points (Figures 4C and 4D). While ALP activity increased in a time-dependent manner both in MC3T3-obif and -cont cells, MC3T3-obif cells showed a significantly higher ALP activity than the MC3T3-cont cells at all points examined (Figure 4C). In contrast, the reduction of *obif *expression caused a significant decrease of ALP activity at all time points examined (Figure 4D). Second, we cultured MC3T3-E1 cells in the differentiation-induction medium until mineralized nodules could be observed. Mineralization was visualized by Alizarin Red staining and quantified by a biochemical assay for calcium. A significantly higher degree of mineralization was obtained in MC3T3-obif cells than in MC3T3-cont cells (Figures 4E-H). A quantitative analysis of calcium by extraction of the calcified mineral also showed a higher calcium content in MC3T3-obif cells (Figure 4M). In contrast, decreased mineralization was observed in MC3T3-sh292 cells and MC3T3-sh301 cells as compared with MC3T3-sh-cont cells (Figures 4I-L and 4N). In addition, we examined the effect of human *OBIF *on mineralization and confirmed that it also promoted mineralization (Additional file 4).

**Figure 5:**
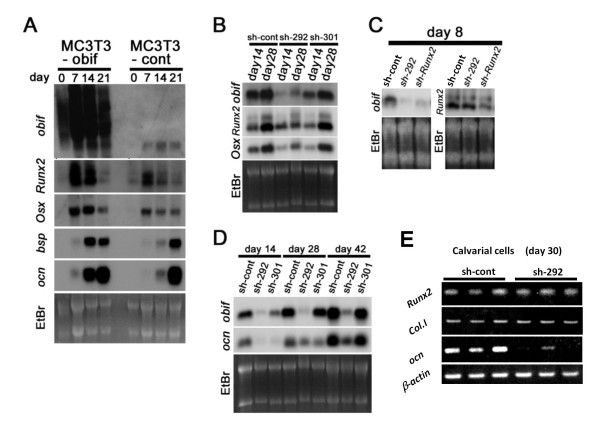
**The effect of *obif *expression on osteoblastic markers**. (A-D) Northern blot analyses of osteoblastic differentiation markers in MC3T3-E1 cells. Comparison between MC3T3-obif cells and MC3T3-control cells (A). The overexpression of *obif *was confirmed in MC3T3-obif cells. Since the coding sequence of *obif *is connected with an IRES sequence in the retrovirus construct, transcripts produced are longer than endogenous *obif *transcripts. The mRNA levels of osteoblastic differentiation markers, in particular *bsp *and *ocn *increased in MC3T3-obif cells. The comparison among control cells (MC3T3-sh147) and *obif *knocked down cells (MC3T3-sh292, -sh301) (B-D). At day 14, *Osx *transcripts decreased in *obif *knocked down cells whereas *Runx2 *transcripts were not influenced by *obif *level (B). At day 28, *Runx2 *was down-regulated in MC3T3-sh292 cells where we could detect only faint levels of *obif *mRNA. In MC3T3-sh*Runx2 *cells, obif was down-regulated whereas *Runx2 *was not affected in MC3T3-sh292 cells at day 8 (C). At day 14 and 28, *ocn *were down-regulated in both MC3T3-sh292 and -sh301 cells (D). At day 42, *ocn *mRNA level in MC3T3-sh301 cells is almost the same as that in the control cells, but significantly lower in MC3T3-sh292 cells. (E) Semi-quantitative RT-PCR of osteoblastic differentiation markers in primary calvarial cells. The data presented are derived from three independent experiments.

Aiming to investigate the effect of *obif *at the molecular level, we examined the expression of osteoblast lineage-associated genes by Northern blot analysis (Figures 5A-D). In *obif *overexpression experiments, the mRNA levels of the osteoblastic differentiation markers increased in MC3T3-obif cells compared with those in MC3T3-cont cells. In particular, the elevations of *bsp *and *ocn *mRNA levels were prominent (Figure 5A). In knockdown experiments, *obif *transcripts were almost completely abolished in MC3T3-sh292 cells and partially ablated in MC3T3-sh301 cells (Figures 5B-D). As early and essential osteoblastic markers, we first examined *Runx2 *and *Osx *mRNA levels (Figure 5B). At day 14, *Osx *transcripts decreased in *obif *knocked down cells whereas *Runx2 *transcripts were not influenced by *obif *levels. At day 28, *Runx2 *was down-regulated in MC3T3-sh292 cells while we could detect only faint evidence of *obif *mRNA. To investigate the relationship between *Runx2 *and *obif*, we established *Runx2 *knocked down MC3T3-E1 cells (MC3T3-sh*Runx2*) and compared with *obif *knocked down cells (Figure 5C). In MC3T3-sh*Runx2 *cells, *obif *was down-regulated whereas *Runx2 *was not affected in MC3T3-sh292 cells at day 8. These results concur with a previous report that suggests *obif *is a downstream gene of *Runx2 *[21]. Furthermore, *obif *promoters contain a number of putative *Runx2 *binding sites in multiple species (Additional file 5). Next, we examined *ocn *mRNA levels in *obif *knocked down cells (Figure 5D). At day 14 and 28, *ocn *was down-regulated in both MC3T3-sh292 and -sh301 cell. At day 42, the *ocn *mRNA level in MC3T3-sh301 cells is almost the same as that in the control cells whereas it is significantly lower in MC3T3-sh292 cells. In primary culture cells, *ocn *mRNA was down-regulated in *obif *knocked-down cells (Figure 5E).

### Extracellular domain of obif can promote osteoblastic differentiation

**Figure 6:**
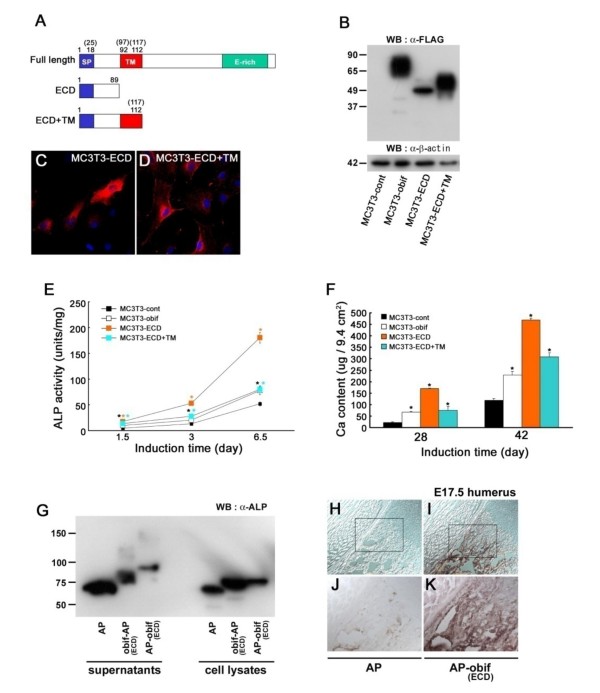
**Extracellular domain of obif can promote osteoblast differentiation**. (A) Schematic representation of partial obif proteins overexpressed in MC3T3-E1 cells. Upper: the full-length obif protein, middle: the partial obif fragment containing the N-terminal extracellular domain (ECD), lower: the partial obif fragment composed of the ECD and the transmembrane domain (TM). Numbers represent amino acid residues of mouse (human) obif proteins. (B-F) MC3T3-E1 cells infected with retroviruses were cultured in the differentiation medium and used for assays. Both of bands of partial obif poteins detected by Western blot were larger than predicted sizes (B). Immunocytochemistry analyses using anti-FLAG antibody (C-D). MC3T3-ECD+TM protein localizes to the plasma membranes whereas a large amount of obif-ECD protein localizes to the cytoplasm. Scale bars = 50 μm. MC3T3-E1 cells infected with retroviruses expressing a full-length obif or its partial proteins showed elevated ALP activities at all time points examined (E). Infection with retroviruses expressing full-length and partial proteins significantly promoted mineral deposition both at day 28 and 42 (F). (G) AP fusion proteins (obif-ECD-AP, AP-obif-ECD) and AP protein were detected by Western blotting analysis using anti-AP antibody. Fusion proteins in supernatants are larger than those in lysates from transfected cells. (H-I) AP-obif (ECD) bound to bone tissues whereas control AP protein did not bind at detectable levels. (J, K) Higher magnifications of the squares in *H *&*I*, respectively. Scale bars = 100 μm.

Obif protein is a single pass transmembrane protein that localizes to the plasma membranes. In order to investigate the mechanism of *obif *function, we made two more retroviral constructs. One expresses the N-terminal extracellular domain (ECD), and the other expresses both the ECD and the transmembrane domain (TM) (Figure 6A). Using retroviruses derived from these constructs, we established cells stably expressing partial obif proteins. Both bands of the partial proteins detected by Western blot were larger than their predicted sizes (Figure 6B). Immunocytochemistry analyses using an anti-FLAG antibody showed different subcellular localizations of these genes (Figures 1F, 6C, and 6D). Proteins including the TM localized to the plasma membranes whereas a large amount of obif-ECD protein localized to the cytoplasm.

Then, we examined the effects of partial obif proteins on the induction of ALP activity and matrix mineralization. MC3T3-ECD and -ECD+TM cells showed strong ALP activities similar to MC3T3-obif cells at all points examined (Figure 6E). Partial obif proteins also stimulated mineralization at days 28 and 42 (Figure 6F). Similar results were observed in experiments using retroviruses expressing partial proteins of human OBIF (Additional file 6).

Next, we generated an obif-fusion protein, consisting of the obif extracellular domain fused to an AP tag. Bands of predicted sizes were detected in lysates from transfected cells by Western blotting using an anti-AP antibody (Figure 6G). On the other hand, in supernatants from transfected cells, bands of larger sizes were detected. When tested for binding to tissue sections and cultured cells, AP-obif (ECD) bound to bone tissues and differentiating MC3T3-E1 cells (Figures 6I, K, and data not shown). In contrast, the control AP protein did not bind at detectable levels (Figures 6H and 6J).

## Discussion

We have isolated and functionally characterized a novel plasma membrane protein, obif. *Obif *is highly expressed in early and late stage osteoblasts of developing mouse embryos, and its transcripts increase during osteoblastic differentiation in several skeletal cell lines. In preosteoblastic MC3T3-E1 cells, differentiation, particularly matrix mineralization, is stimulated when *obif *is overexpressed, and inhibited when *obif *is knocked down. Furthermore, we showed that the extracellular domain of obif protein can promote MC3T3-E1 differentiation like the full-length form of obif, suggesting that obif functions in a ligand-like manner.

Although *obif *was originally isolated as a molecule that is up-regulated during ATDC5 cell differentiation, we could not detect clear *obif/*obif expression in chondrocyte-lineage cells in mouse skeletal tissues. This seems due to *obif *up-regulation by cells surrounding the cartilage nodules during ATDC5 differentiation.

Osteoblastogenesis is a multistep process temporally controlled by transcription factors and secreted growth factors. The centerpiece of the transcriptional control of osteoblast differentiation is the *Runx2*-dependent pathway. *Runx2*-deficient mice have a cartilaginous skeleton without any osteoblasts, as osteoblast differentiation is arrested as early as E12.5 [6]. *Osterix*, a zinc finger-containing protein that is known to be downstream of *Runx2*, is expressed in osteoblast progenitors, and mice without *Osterix *also lack mature osteoblasts [7]. Although other transcription factors, Dlx5/6, Msx1/2, AP-1 families, twist, and ATF4, have been reported to be involved in osteoblastic differentiation, exact roles of these factors and their relationship with Runx2 remain to be elucidated [22-27]. A recent report suggests that *obif (tmem119) *is one of the molecules downstream of *Runx2 *[21]. Our results are consistent with this report. However, *obif *expression is observed as early as *Runx2 *expression in developing limb buds and during the differentiation of MC3T3-E1 cells. Therefore, the genetic and cell biological relationships between *obif *and *Runx2 *will need future studies, including analysis of *obif *and *Runx2 *mutant mice.

In mouse embryos, cells expressing *obif *are observed in the periosteum, bone collars, and trabecular bones. The expression pattern of *obif *suggests that *obif *functions in osteoblastic development. In the multiple cell line models we used, endogenous *obif *is markedly upregulated during osteoblastic differentiation. These expression patterns strongly suggest that *obif *functions in osteoblastogenesis. In committed preosteoblastic MC3T3-E1 cells, *obif *exerts critical roles both in ALP induction and in matrix mineralization. In *obif *overexpression and knockdown experiments, cell proliferation is mildly affected but the effect of *obif *expression levels on differentiation is more significant. Thus we propose that obif may play a role in both proliferation and differentiation, but the primary effect of *obif *is on differentiation. When *obif *is knocked down in MC3T3-E1 cells, mRNA levels of *Osx *and *ocn *decrease proportionally to the *obif *transcripts, although *Runx2 *is not significantly affected. And in a similar experiment using primary calvarial cells, the level of *ocn *transcript decreases in cells in which obif was knocked down. These results suggest that obif protein is involved in the late differentiation of osteoblast-lineage cells.

Partial proteins including the ECD of both human and mouse obif stimulated osteoblastic differentiation in MC3T3-E1 cells as well as the full-length obif protein. These results suggest that the ECD is sufficient to stimulate osteoblastic differentiation, and the physiological function of obif is mediated by cell-cell or cell-matrix interaction as a ligand. The binding activity of AP-obif (ECD) to bone tissues and differentiating MC3T3-E1 cells supports the hypothesis that an *obif *receptor-like molecule exists in bone tissues and that *obif *functions as a ligand. Further investigation of the functional mechanisms of *obif *may shed light on a new signaling mechanism in osteoblast and chondrocyte differentiation.

## Conclusions

We have isolated and functionally characterized a novel plasma membrane protein, obif. *Obif *is highly expressed in early and late stage osteoblasts of developing mouse embryos, and its transcripts increase during osteoblastic differentiation in several skeletal cell lines. In preosteoblastic MC3T3-E1 cells, differentiation, particularly matrix mineralization, is stimulated when *obif *is overexpressed, and inhibited when *obif *is knocked down. Furthermore, we showed that the extracellular domain of obif protein can promote MC3T3-E1 differentiation like the full-length form of obif, suggesting that obif functions in a ligand-like manner.

## Methods

### Reagents

Cell culture medium, α-modified minimum essential medium (α-MEM), Dulbecco's modified Eagle's medium (DMEM), a 1:1 mixture of DMEM and Ham's F-12 medium, ascorbic acid, β-glycerophosphate, and ITS Liquid Media Supplement were purchased from Sigma. Fetal bovine serum (FBS) was purchased from JRH Bioscience (Nichirei, Japan). The following primary antibodies were used: anti-FLAG M2 (Sigma), anti-β-actin clone AC-74 (Sigma), anti-collagen type I (Santa Cruz). Wnt3a-conditioned medium was kindly provided by Dr. Shin Yonehara (Kyoto University). Recombinant human BMP2 was provided by Osteopharma Inc. (Osaka). *Obif *siRNA (Stealth™ RNAs) duplexes were chemically synthesized by Invitrogen. The *obif *siRNA sequences were as follows: siRNA-1, GCUCGCUGACCUUCCUCAUCAUGUU; siRNA-2, UCGCUGACCUUCCUCAUCAUGUUCA; siRNA-3, CCUUCCUCAUCAUGUUCAUAGUCUG.

### Anti-obif antibody production

By using PCR, a cDNA encoding a C-terminal portion of mouse obif (residues 113-280; obif-C) was amplified and subcloned into pGEX4T-1 (Amersham Biosciences). The fusion was expressed in *Escherichia coli *strain DH5α and purified with glutathione Sepharose 4B (Amersham Biosciences) according to the manufacturer's instructions. An antibody against obif was obtained by immunizing rabbits with the purified GST-obif-C (MBL, Japan). The rabbit antisera against obif-C was pre-absorbed with GST-Sepharose and affinity purified with an immunizing fusion protein-bound Sepharose column.

### Cell culture

ATDC5, MC3T3-E1, ST2, and C2C12 cells were obtained from the RIKEN Cell Bank (Ibaraki, Japan). The ATDC5 cells were cultured in a maintenance medium of a 1:1 mixture of DMEM and Ham's F-12 medium, and 5% FBS. MC3T3-E1 cells and ST2 cells were cultured in α-MEM supplemented with 10% FBS. C2C12 cells were cultured in DMEM containing 10% FBS. Primary mouse calvarial cells were dissected from 2-day old pups. Calvarial cells were isolated by four sequential 15-minute digestions in collagenase/dispase solution (Roche 269638) at 37°C. Fractions 2-4 were collected, resuspended in media and plated. Freshly harvested cells of less than 4 passages were used for all experiments.

### Microarray gene expression profiles

RNA was harvested and purified with Trizol according to manufacturer's protocol (Invitrogen). Preparation of cRNA and hybridization of probe arrays were performed according to the protocols of the manufacturer using the GeneChip Mouse Genome 430 2.0 Array (Affymetrix, Santa Clara, CA).

### Production of recombinant retrovirus and infection

The retroviral vector expressing mouse or human obif was constructed in the pBMN-I-GFP vector that was kindly provided by Dr. Gary Nolan (Stanford University). In brief, the full- or partial-length coding regions of mouse or human *obif *cDNA that were added to the FLAG tag at the C-terminus were inserted into the multicloning site of pBMN-I-GFP. PCR were performed using FANTOM clone [28] cDNA (#BC025600) or cDNA produced from Human Fetal Brain Total RNA (purchased from Clontech). Reduction of obif expression was achieved through the retroviral infection of cells with short hairpin RNAs directed against the *obif *mRNA, using pSUPER.retro.neo+GFP (pSUP) plasmids (Oligoengine). 19-nt target sequences were selected in the mouse *obif and Runx2 *cDNA coding sequences as follows: *obif *sh292, 5'-CGCTGACCTTCCTCATCAT-3' (292-310); *obif *sh301, 5'-TCCTCATCATGTTCATAGT-3' (301-319); *Runx2*, 5'-CCACTTACCACAGAGCTAT-3' (857-875). Each one was used to design a 60-nt oligo, which was subcloned between the *BglII *and *HindIII *restriction sites of the pSUP vector according to the manufacturer's instructions. As a control, the same set of experiments was performed using an irrelevant sequence (Dharmacon Research). To produce the retroviral particles, the plasmid DNA was transfected along with a helper plasmid into a subline of the 293T cell line; supernatant was collected every day starting at 24 hr posttransfection for 3 days. For infection, cells plated at low density were incubated in virus-conditioned medium for 1-2 days in the presence of 8 μg/ml polybrene. Virus-conditioned medium was used in a 1:1 dilution with normal growth medium. In the case of infection with retroviruses expressing shRNA, infected cells were selected based on their resistance to G418.

### Western blot analysis

Cells were harvested in lysis buffer (50 mM Tris-HCL [pH7.5], 0.15 M NaCl, containing 1% NP-40, 1 μM sodium orthovanadate). After sonication, cell debris was removed by centrifugation, and the supernatants were collected. Equal amounts of total proteins were then prepared for Western blot analysis according to a previously described protocol [29]. The primary antibody dilutions were 1:1000 (anti-FLAG M2), 1:500 (anti-obif), and 1:5000 (anti-β-actin).

### Subcellular localization analysis

Stably infected cells were used for localization analysis according to a previously described protocol [29]. The primary antibodies were used at dilutions of 1:500 (anti-FLAG M2) and 1:250 (anti-obif). For nuclear staining, we added 4,6, -diamidino-2-phenylindole dihydrochloride (DAPI, Sigma) at a dilution of 1:1000 in the secondary antibody solution.

### Subcellular fractionation

Cells were harvested in ice-cold buffered sucrose (0.25 M. sucrose-10 mM Tris-HCl buffer [pH 7.5]) and whole-cell lysates prepared by sonication were centrifuged in a swinging bucket rotor at 80 × g for 5 min at 4°C. Then the supernatant was spun at 12,000 × g for 10 min. The resultant pellet was designated the mitochondrial fraction. The postmitochondrial supernatant was then subjected to centrifugation at 100,000 × g for 90 min in a Beckman TLA120.2 rotor at 4°C. The high-spin pellet was designated the microsomal fraction, and the supernatant was designated the cytoplasmic fraction.

### *In situ *hybridization and Northern blot analysis

Mouse *obif *(#BC025600), *Sox9 *(#4933413I11), and *collagen1 *(#D930048E22) cDNAs obtained from the RIKEN FANTOM2 collection were used to prepare antisense probes. A 540 bp fragment of mouse *collagen2 *and a 520 bp fragment of mouse *collagen10 *were obtained by RT-PCR and subcloned into the pGEM-T Easy vectors (Promega). Mouse *osteopontin *and *osteocalcin *cDNAs were kindly provided by Dr. Toshihisa Komori (Nagasaki University, Japan). Whole mount *in situ *hybridization and section *in situ *hybridization were performed as described previously [30]. Northern blot hybridization was performed as described previously [31]. The entire protein coding regions of *obif, bsp*, a 1-kb fragment of *Runx2 *cDNA (nucleotides 280-1281), a 0.5-kb fragment of *Osterix *cDNA (nucleotides 115-611), and a 0.47-kb fragment of *ocn *cDNA (nucleotides 1-470) were used as radiolabeled probes.

### Immunohistochemistry

We followed the immunostaining procedures described previously [32]. The specimens were observed under a laser confocal microscope (LSM510, Carl Zeiss) or a fluorescent microscope (Axioskop 2 Plus, Carl Zeiss).

### Proliferation assay

Cells were plated at a density of 3 × 10^3 ^cells/cm^2 ^and cultured in growth medium. At each time point, cells were harvested and counted using a haemocytometer. Duplicate measurements were performed on three independent wells for each time point.

### Osteoblastic differentiation

MC3T3-E1 cells, ST2 cells: Cells were plated in 6-well or 12-well plates in triplicate at a density of 2.5 × 10^4 ^cells/cm^2 ^and were cultured in growth medium. One day after the cells reached confluence, they were cultured in differentiation medium (α-MEM containing 50 ug/ml ascorbic acid and 2 mM β-glycerophosphate) or Wnt3a- conditioned medium that was used at a 1: 3 dilution with normal growth medium. Determination of ALP activity, Alizarin Red S staining and Ca^2+ ^accumulation was done as described elsewhere [33,34]. C2C12 cells: For BMP2 induction, cells were cultured in medium supplemented with 2.5% FBS and 300 ng/ml recombinant human BMP2.

### Chondrogenic differentiation

After ATDC5 cells were plated in 6-well or 12-well plates in triplicate at a density of 2.5 × 10^4 ^cells/cm^2^, differentiation induction and evaluation of matrix proteoglycan synthesis were performed as described elsewhere [35].

### Semi-quantitative RT-PCR

One μg samples of RNA were subjected to reverse transcription with a random hexamer as a primer, and semi-quantitative PCR was performed for each gene using the following primers: for mouse *runx2*, 5'-AAATGCCTCCGCTGTTATGAA (sense primer) and 5'-GCTCCGGCCCACAAATCT (antisense primer); for mouse *collagen 1*, 5'-CCCAAGGAAAAGAAGCACGTC (sense primer) and 5'-AGGTCAGCTGGATAGCGACATC (antisense primer); for mouse *ocn*, 5'-CCGGGAGCAGTGTGAGCTTA (sense primer) and 5'-AGGCGGTCTTCAAGCCATACT (antisense primer); and for mouse *β-actin *5'-CGTGCGTGACATCAAAGAGAA (sense) and 5'-TGGATGCCACAGGATTCCAT (antisense). PCR conditions were 28 cycles of 94°C for 20 sec, 55°C for 30 sec, and 72°C for 30 sec. Post PCR, each sample was subjected to electrophoresis on a 2% agarose gel and visualized by ethidium bromide staining.

### Statistical analysis

Data were examined for distribution, variance homogeneity (F-test) and analyzed using Student's unpaired *t*-test (2 tailed) for all analyses. P < 0.05 was considered statistically significant. Unless otherwise specified, all data are presented as mean ± SEM or mean + SE.

### Production of the AP tag-obif fusion protein

We followed the procedures described previously [36]. Briefly, AP fusion proteins were produced by inserting the extracellular domain of obif into the APtag-5 vector that was kindly provided by Dr. Mitsuharu Hattori (Nagoya City University). After producing conditioned medium from transiently transfected 293T cells, we spun out debris and filtered (0.45-μm pore size) the supernatant.

### *In situ *analysis of AP tag-obif binding to tissue sections

We washed the sections in HBS and in HBAH buffer. AP fusion proteins were added and incubated at room temperature for 90 min. After a 15 min incubation in preheated HBS, in a 65° water bath, BCIP/NBT substrate was added.

## Competing interests

The authors declare that they have no competing interests.

## Authors' contributions

TK and TF designed the project. TK carried out the molecular, cell biological and immunohistochemical studies. KM and KT participated in the cell biological study. TM and TK carried out microarray analysis. TK and TF wrote the manuscript. HY and TF supervised the project.

## Supplementary Material

Additional file 1 ***obif *is conserved among species**. The deduced amino acid sequences of chicken, mouse, rat, and human obif proteins. GenBank accession numbers for the sequences are chicken, XP_415183.1; mouse, BK006092; rat, NP_001100625.1; human, AAQ88755.1. All of them contain the N-terminal signal peptide, a single transmembrane domain, and a glutamic acid-rich region (E-rich). Black boxes indicate potential O-glycosylation sites conserved among species. Click here for file

Additional file 2 **Verification of overexpression of obif protein by immunostaining**. MC3T3-E1 and ATDC5 cells were infected with retroviruses expressing both obif and GFP (MC3T3-obif) (A-C), (ATDC5-obif) (G-I) or expressing GFP only (MC3T3-cont) (D-F), (ATDC5-cont) (J-L), Cells were stained with anti-obif antibody (red) and nuclei were stained with DAPI (blue) (C, F, I, L). Click here for file

Additional file 3 **Verification of knock down of obif protein by Western blot analyses**. (Left) Western blot analysis of cell extracts from MC3T3-obif and ATDC5-obif. To knock obif down, each of three siRNAs designed against obif and a negative-control siRNA were transfected into cell lines. Non-transfected cells were also electrophoresed. Blots were probed with anti-obif antibody. (Right) Western blot analysis of MC3T3-obif infected with retroviruses expressing control sh-cont, sh301, and sh292. Blots were probed with anti-obif antibody. Sh292 sequence overlaps siRNA-1 and siRNA-2 sequences, and sh301 sequence overlaps siRNA-3 sequence. Suppressive effect of sh292 is significantly stronger than that of sh301. Click here for file

Additional file 4 **Calcium contents observed in MC3T3-E1 cells expressing human obif and control cells at days 18 and 26**. Calcium contents were significantly higher in MC3T3-hOBIF cells than in MC3T3-cont cells both at day 18 and 26. Click here for file

Additional file 5 **Comparative analysis of *obif *gene promoter**. Schematic illustration of chick, mouse, rat, and human *obif *promoter. Putative *Runx2 *binding sites are indicated by open triangle. Click here for file

Additional file 6 **Retroviruses expressing human *OBIF *exhibited a similar effect on mineralization of MC3T3-E1 cells as those infected with mouse *obif *retroviruses**. Infection with retroviruses expressing full-length and partial human OBIF significantly promoted mineral deposition at day 33. Click here for file
