# Supplementary material for: Isolation and characterization of a novel plasma membrane protein, osteoblast induction factor (obif), associated with osteoblast differentiation
Source: BMC Dev Biol. 2009 Dec 21;9:70. doi: 10.1186/1471-213X-9-70 (PMC2805627; doi:10.1186/1471-213X-9-70)
Supplement: Additional file 1 — obif is conserved among species. The deduced amino acid sequences of chicken, mouse, rat, and human obif proteins. GenBank accession numbers for the sequences are chicken, XP_415183.1; mouse, BK006092; rat, NP_001100625.1; human, AAQ88755.1. All of them contain the N-terminal signal peptide, a single transmembrane domain, and a glutamic acid-rich region (E-rich). Black boxes indicate potential O-glycosylation sites conserved among species. [file 1471-213X-9-70-S1.PDF]

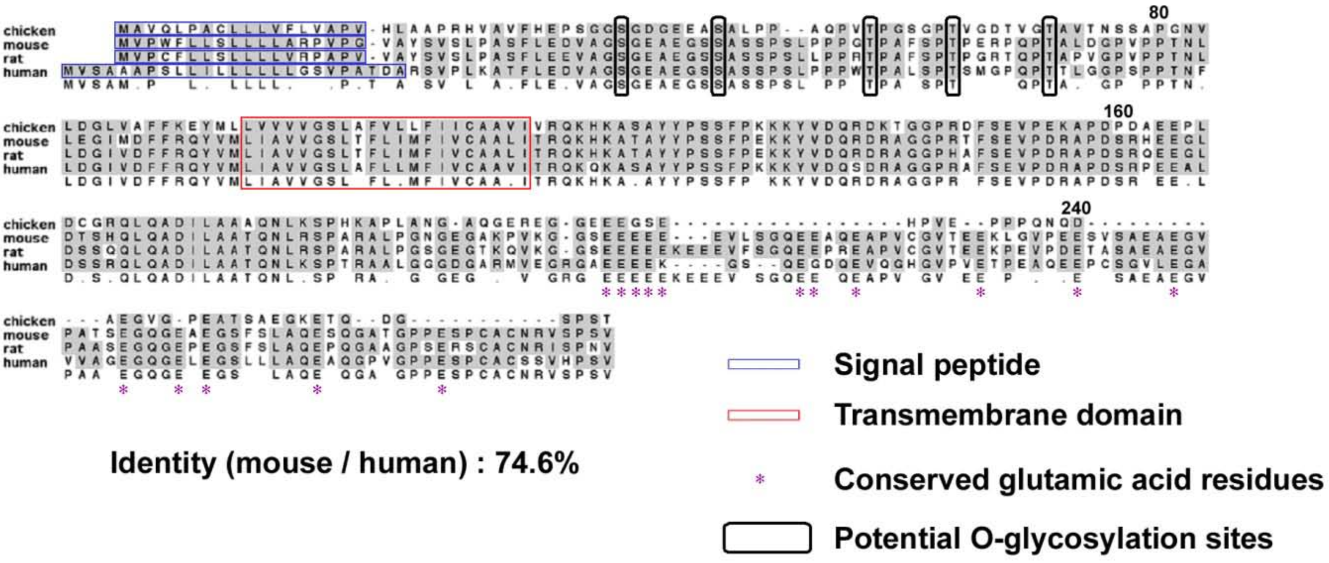
chicken
mouse
rat
human
80
160
240
Signal peptide
Transmembrane domain
Conserved glutamic acid residues
Potential O-glycosylation sites
Identity (mouse / human) : 74.6%
