# Supplementary figures and images for: Isolation and characterization of a novel plasma membrane protein, osteoblast induction factor (obif), associated with osteoblast differentiation
Source: BMC Dev Biol. 2009 Dec 21;9:70. doi: 10.1186/1471-213X-9-70 (PMC2805627; doi:10.1186/1471-213X-9-70)

MC3T3  
-obif

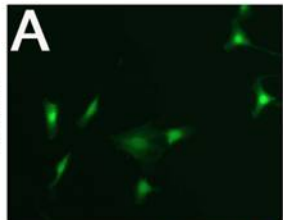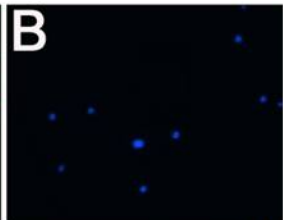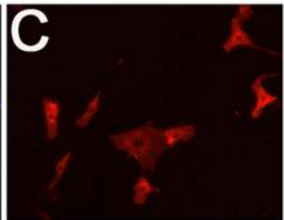

MC3T3  
-cont

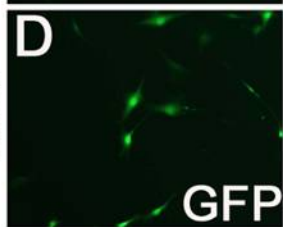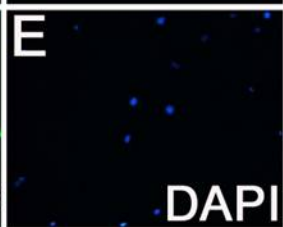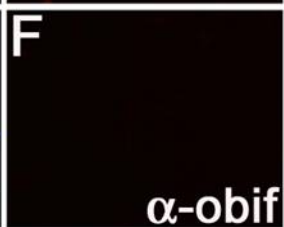

ATDC5  
-obif

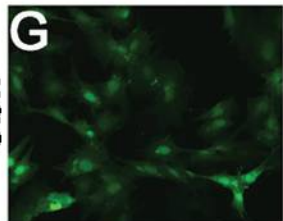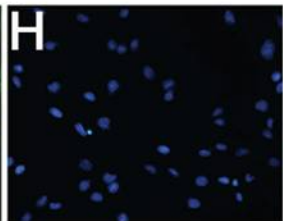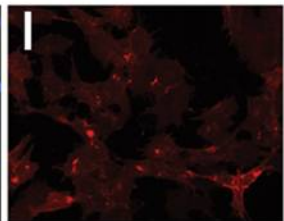

ATDC5  
-cont

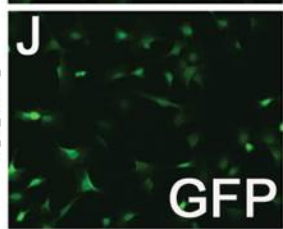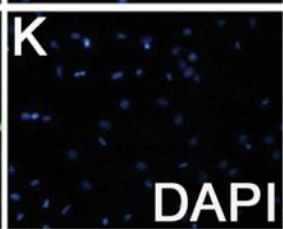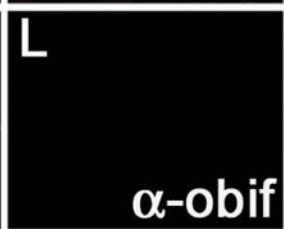

Supplement: Additional file 2 — Verification of overexpression of obif protein by immunostaining. MC3T3-E1 and ATDC5 cells were infected with retroviruses expressing both obif and GFP (MC3T3-obif) (A-C), (ATDC5-obif) (G-I) or expressing GFP only (MC3T3-cont) (D-F), (ATDC5-cont) (J-L), Cells were stained with anti-obif antibody (red) and nuclei were stained with DAPI (blue) (C, F, I, L). [file 1471-213X-9-70-S2.PDF]

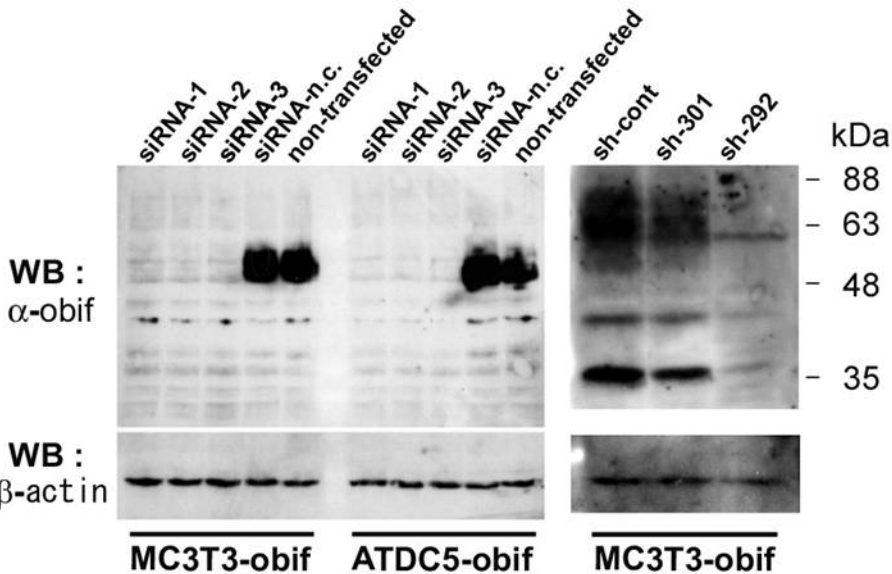

Supplement: Additional file 3 — Verification of knock down of obif protein by Western blot analyses. (Left) Western blot analysis of cell extracts from MC3T3-obif and ATDC5-obif. To knock obif down, each of three siRNAs designed against obif and a negative-control siRNA were transfected into cell lines. Non-transfected cells were also electrophoresed. Blots were probed with anti-obif antibody. (Right) Western blot analysis of MC3T3-obif infected with retroviruses expressing control sh-cont, sh301, and sh292. Blots were probed with anti-obif antibody. Sh292 sequence overlaps siRNA-1 and siRNA-2 sequences, and sh301 sequence overlaps siRNA-3 sequence. Suppressive effect of sh292 is significantly stronger than that of sh301. [file 1471-213X-9-70-S3.PDF]

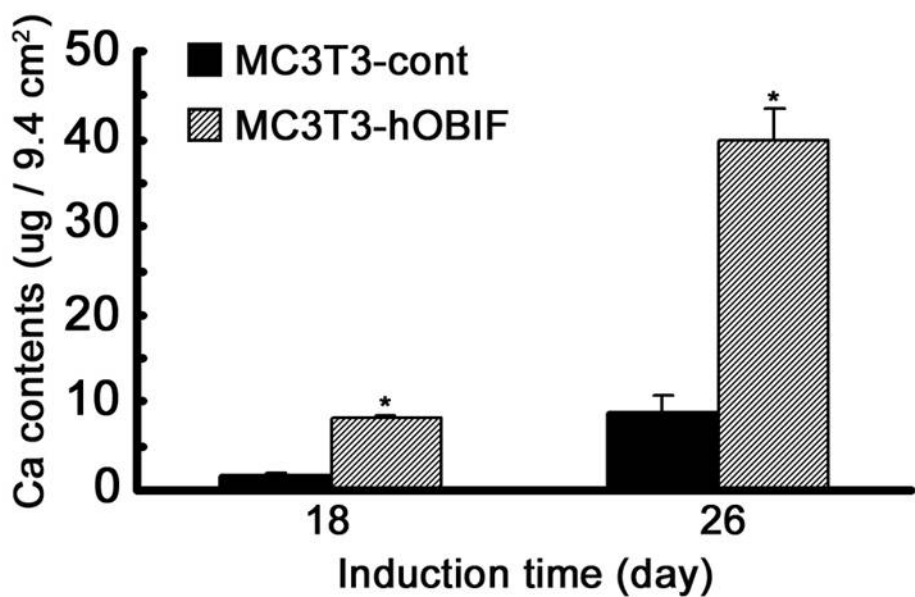

Supplement: Additional file 4 — Calcium contents observed in MC3T3-E1 cells expressing human obif and control cells at days 18 and 26. Calcium contents were significantly higher in MC3T3-hOBIF cells than in MC3T3-cont cells both at day 18 and 26. [file 1471-213X-9-70-S4.PDF]

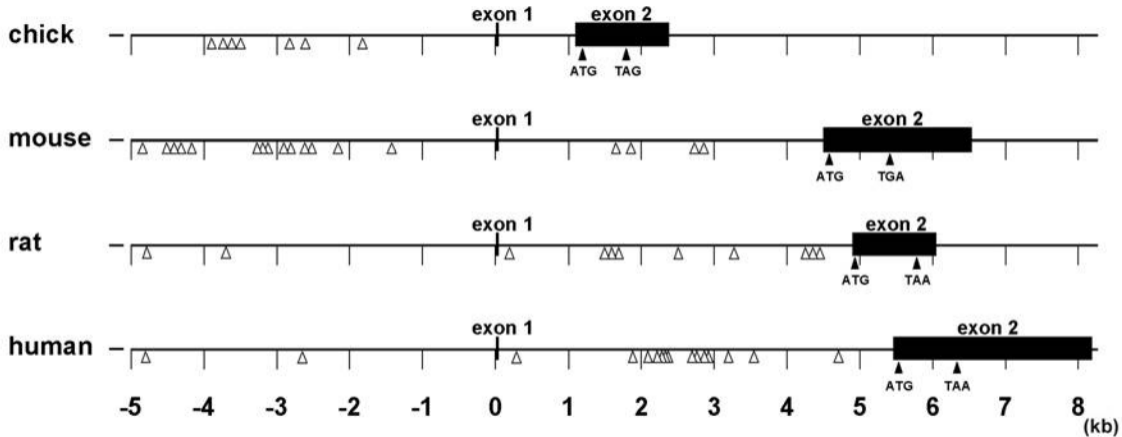

Supplement: Additional file 5 — Comparative analysis of obif gene promoter. Schematic illustration of chick, mouse, rat, and human obif promoter. Putative Runx2 binding sites are indicated by open triangle. [file 1471-213X-9-70-S5.PDF]

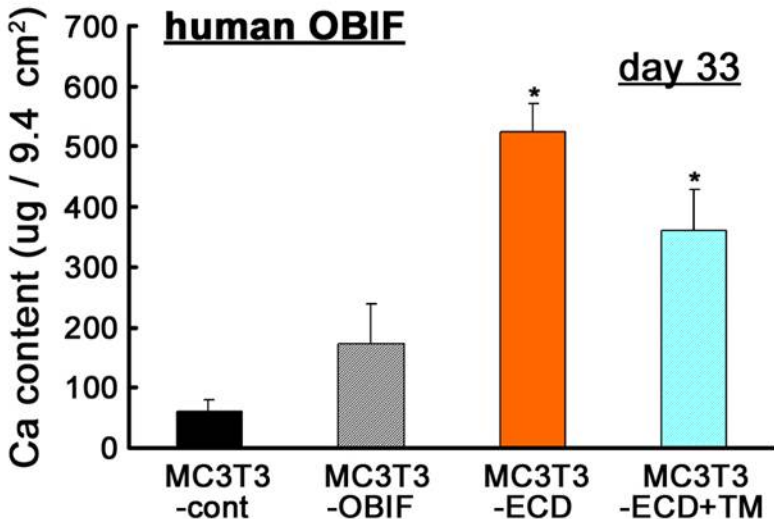

Supplement: Additional file 6 — Retroviruses expressing human OBIF exhibited a similar effect on mineralization of MC3T3-E1 cells as those infected with mouse obif retroviruses. Infection with retroviruses expressing full-length and partial human OBIF significantly promoted mineral deposition at day 33. [file 1471-213X-9-70-S6.PDF]
